# Induction of the nicotinamide riboside kinase NAD^+^ salvage pathway in a model of sarcoplasmic reticulum dysfunction

**DOI:** 10.1186/s13395-019-0216-z

**Published:** 2020-02-19

**Authors:** Craig L. Doig, Agnieszka E. Zielinska, Rachel S. Fletcher, Lucy A. Oakey, Yasir S. Elhassan, Antje Garten, David Cartwright, Silke Heising, Ahmed Alsheri, David G. Watson, Cornelia Prehn, Jerzy Adamski, Daniel A. Tennant, Gareth G. Lavery

**Affiliations:** 1 Institute of Metabolism and Systems Research, University of Birmingham, 2nd Floor IBR Tower, Edgbaston, Birmingham, B15 2TT UK; 2 Centre for Endocrinology, Diabetes and Metabolism, Birmingham Health Partners, Birmingham, UK; 3 Strathclyde Institute of Pharmacy and Medical Sciences, Hamnett Wing John Arbuthnott Building, Glasgow, G4 0RE UK; 4 Research Unit of Molecular Endocrinology and Metabolism, Helmholtz Zentrum Munchen GmbH, Ingolstadter Landstrasse 1, D-85764 Neuherberg, Germany; 5 Lehrstuhl für Experimentelle Genetik, Technische Universität München, Freising, Germany; 6 Department of Biochemistry, Yong Loo Lin School of Medicine, National University of Singapore, Singapore, 117593 Singapore; 7 MRC-ARUK Centre for Musculoskeletal Ageing Research, University of Birmingham, Birmingham, UK

**Keywords:** Hexose-6-phosphate dehydrogenase, Endoplasmic/sarcoplasmic reticulum, Skeletal muscle, Nicotinamide riboside, Nicotinamide adenine dinucleotide

## Abstract

**Background:**

Hexose-6-Phosphate Dehydrogenase (H6PD) is a generator of NADPH in the Endoplasmic/Sarcoplasmic Reticulum (ER/SR). Interaction of H6PD with 11β-hydroxysteroid dehydrogenase type 1 provides NADPH to support oxo-reduction of inactive to active glucocorticoids, but the wider understanding of H6PD in ER/SR NAD(P)(H) homeostasis is incomplete. Lack of H6PD results in a deteriorating skeletal myopathy, altered glucose homeostasis, ER stress and activation of the unfolded protein response. Here we further assess muscle responses to H6PD deficiency to delineate pathways that may underpin myopathy and link SR redox status to muscle wide metabolic adaptation.

**Methods:**

We analysed skeletal muscle from H6PD knockout (H6PDKO), H6PD and NRK2 double knockout (DKO) and wild-type (WT) mice. H6PDKO mice were supplemented with the NAD^+^ precursor nicotinamide riboside. Skeletal muscle samples were subjected to biochemical analysis including NAD(H) measurement, LC-MS based metabolomics, Western blotting, and high resolution mitochondrial respirometry. Genetic and supplement models were assessed for degree of myopathy compared to H6PDKO.

**Results:**

H6PDKO skeletal muscle showed adaptations in the routes regulating nicotinamide and NAD^+^ biosynthesis, with significant activation of the Nicotinamide Riboside Kinase 2 (NRK2) pathway. Associated with changes in NAD^+^ biosynthesis, H6PDKO muscle had impaired mitochondrial respiratory capacity with altered mitochondrial acylcarnitine and acetyl-CoA metabolism. Boosting NAD^+^ levels through the NRK2 pathway using the precursor nicotinamide riboside elevated NAD^+^/NADH but had no effect to mitigate ER stress and dysfunctional mitochondrial respiratory capacity or acetyl-CoA metabolism. Similarly, H6PDKO/NRK2 double KO mice did not display an exaggerated timing or severity of myopathy or overt change in mitochondrial metabolism despite depression of NAD^+^ availability.

**Conclusions:**

These findings suggest a complex metabolic response to changes in muscle SR NADP(H) redox status that result in impaired mitochondrial energy metabolism and activation of cellular NAD^+^ salvage pathways. It is possible that SR can sense and signal perturbation in NAD(P)(H) that cannot be rectified in the absence of H6PD. Whether NRK2 pathway activation is a direct response to changes in SR NAD(P)(H) availability or adaptation to deficits in metabolic energy availability remains to be resolved.

## Background

Our understanding of the control of NADP(H) redox homeostasis within muscle sarcoplasmic reticulum (SR) and its influence over cellular metabolism is developing [1–4]. A major enzyme that reduces NADP^+^ within the SR lumen is hexose-6-phosphate dehydrogenase (H6PD) which oxidises glucose-6-phosphate derived from glycolysis to generate NADPH [5, 6]. A known function of H6PD is its physical interaction with 11β-hydroxysteroid dehydrogenase type 1 (11β-HSD1) in the SR lumen [7]. H6PD NADPH generation supports 11β-HSD1 oxo-reductase activity for glucocorticoid production [3]. The importance of the H6PD-11β-HSD1 interaction is clear in humans with ‘apparent’ cortisone reductase deficiency (ACRD) due to inactivating mutations in the H6pd gene [8, 9]. In ACRD, the lack of H6PD and its associated NADPH production switches 11β-HSD1 activity towards glucocorticoid oxidation (and therefore NADP^+^ reduction) resulting in increased tissue glucocorticoid clearance and relative insensitivity. Affected individuals manifest disease as a function of increasing hypothalamic-pituitary-adrenal axis activity and resultant excess adrenal androgen production [10]. H6PD knockout (H6PDKO) mice reflect this biochemistry, manifesting an ACRD-like phenotype, showing tissue glucocorticoid insensitivity and HPA axis activation [6]. Liver and adipose tissues demonstrate phenotypes associated with relative glucocorticoid insensitivity. The liver has impaired ability to stimulate gluconeogenesis, with increased glycogen synthesis rates, while adipose tissue has impaired ability to store and mobilise lipid [11, 12].

Skeletal muscle of H6PD knockout mice develop a progressively deteriorating myopathy associated with large intrafibrillar membranous vacuoles, abnormal sarcoplasmic reticulum (SR) structure, dysregulated expression of SR proteins involved in calcium metabolism and switching of type II to type I fibers and impaired force generation [4]. Associated with myopathy is ER stress and activation of the unfolded protein response- partially attributed to altered SR lumen protein folding capacity [2, 4]. Metabolically H6PDKO demonstrate dysfunction in glucose homeostasis, characterised by fasting hypoglycaemia, increased skeletal muscle insulin sensitivity (particularly in type II fibre-rich muscles) and increased glycogen content [4, 13]. Importantly, H6PDKO myopathy is independent of the role of 11β-HSD1 and glucocorticoids [13].

It has been proposed that H6PD contributes to ER/SR redox balance beyond regulation of 11β-HSD1 activity. This is evident through both its effects on physiological glucose metabolism, as well as adrenal steroidogenic enzyme activity [14]. Less clear is the interaction and contribution of H6PD and ER/SR NADP(H) to whole cell redox and cellular energy metabolism. The major source of cellular NADPH is reduction of NADP^+^ through the pentose phosphate pathway, with NADP^+^ synthesised from NAD^+^ via NAD^+^ kinase [15]. While muscle has the capacity to co-opt multiple pathways for NAD(P)(H) biosynthesis, it largely relies on salvage of vitamin B3 precursors, chiefly nicotinamide (NAM) which can be recycled back to NAD^+^ via NAM phosphoribosyltransferase [16].

We wanted to investigate primary pathways responding to the perturbations in SR NADP(H) redox due to H6PD deficiency using unbiased and targeted metabolomics and existing transcriptional data [4]. We show that in young mice, prior to overt phenotypic presentation, H6PDKO remodels NAD(P)(H) biosynthesis, particularly through the nicotinamide riboside kinase 2 (NRK2) pathway. H6PD deficiency also results in upregulation of pathways to acetyl-CoA production leading to accumulation of short chain acylcarnitines, elevated acetylation of mitochondrial proteins and impaired mitochondrial fatty acid oxidation. Acetylation of the mitochondrial proteome is associated with depression in energy production, and can be countered through the activity of the NAD^+^-dependent deacetylase SIRTs [17, 18]. Taking advantage of elevated NRK2 expression we augmented NAD^+^ availability in H6PDKO mice by supplementing the precursor nicotinamide riboside (NR). Similarly H6PD/NRK2 double KO mice were examined as a means to further limit NAD^+^ availability. While each approach was able to modulate NAD^+^ metabolism, neither was able to rescue or exacerbate mitochondrial dysfunction or myopathy. These findings reveal a complex metabolic response to changes to muscle SR NADP(H) redox status and associated impairment in mitochondrial energy metabolism.

## Materials and methods

### Animal care, mouse strains, storage

All mice (C57/BL6J background) were group housed according to sex in a standard temperature (22 °C) and humidity-controlled environment with 12:12-h light:dark cycle. Nesting material was provided and mice had ad libitum access to water and standard chow. This study used global knockout of H6PD, these phenocopy muscle specific knockout mice [13, 19]. Studies used both male and female mice. During the generation of data there were no sex specific differences observed. Mice were sacrificed using schedule one cervical dislocation Collections were all performed at 10-11 am. For collection of serum, murine blood was obtained by cardiac puncture and immediately centrifuged at 1000 x g in heparin-coated tubes. Serum was transferred to cryotubes and snap-frozen.

Nicotinamide riboside chloride was resuspended in phosphate buffered saline (PBS) and sterile filtered. Vehicle control treatment was sterile PBS alone. Intraperitoneal injections of Nicotinamide Riboside (400 mg/kg) were given twice daily, (10 am and 3 pm) for 4 days before cervical dislocation and skeletal muscle bed collection on Day 5 (10 am), tissues were flash frozen, fixed or for high resolution respirometry stored in BIOPS buffer. Heterozygotes of H6PDKO and NRK2KO were bred to generate H6PD-NRK2 KO mice. Mice were utilised between 8 and 12 weeks of age. Experiments were consistent with current UK Home Office regulations in accordance with the UK Animals Scientific Procedures Act 1986.

### RNA extraction and qRT-PCR

Total RNA was extracted from skeletal muscle tissue using TRI-reagent (Invitrogen). RNA quality was determined by visualisation on a 1.5% agarose gel and quantity was measured by nanodrop absorbance at 260 nm. Reverse transcription was conducted using 1 μg RNA not DNAse treated that was incubated with 250uM random hexamers, 5.5 mM MgCl_2_, 500uM dNTPs, 20 units RNase inhibitor 63 units multi-scribe reverse transcriptase and 1x reaction buffer. Reverse transcription was performed on a thermocycler set at the following conditions: 25 °C 10 min, 48 °C for 30 min before the reaction was terminated by heating to 98 °C for 5 min. cDNA levels were determined using an ABI7500 system (Applied Biosystems), reactions were conducted in a 384-well plate in single-plex format. Primers and probes were purchased as Assay on Demand (FAM) products (Applied Biosystems), predesigned to cross exon boundaries to preclude genomic DNA measurement. Total reaction volumes used were 10ul containing Taqman Universal PCR mix (Applied Biosystems). All reactions were normalised to 18 s rRNA (VIC) (Applied Biosystems). The real-time PCR reaction was performed at the following conditions: 95 °C for 10 min then 40 cycles of 95 °C for 15 s and 60 °C for 1 min. Data were collected as Ct values and used to obtain deltaCt (dCt) values and expressed as fold change ±standard error of the mean (SEM).

### Mitochondrial DNA (mtDNA) copy number

DNA was isolated using phenol-chloroform and washed in Ethanol before quantification using nanodrop. DNA from all samples was diluted to 50 ng/μl and subject to real-time PCR using SYBRgreen master mix (Applied Biosystems) using the following primers specific for nuclear and mitochondrial DNA.

Nuclear DNA: TERT Forward CTAGCTCATGTGTCAAGACCCTCTT Reverse GCCAGCACGTTTCTCTCGTT, Mitochondrial DNA: DLOOP Forward AATCTACCATCCTCCGTGAAACC Reverse TCAGTTTAGCTACCCCCAAGTTTAA, ND4 Forward AACGGATCCACAGCCGTA Reverse AGTCCTCGGGCCATGATT.

### Western blotting

Protein lysates were collected in RIPA buffer (50 mmol/l Tris pH 7.4, 1% NP40, 0.25% sodium deoxycholate, 150 mmol/l NaCl, 1 mmol/l EDTA), protease & phosphatase inhibitor cocktail (Roche, Lewes, U.K.), stored at − 80 °C (> 30 min), defrosted on ice and centrifuged at 4 °C (10mins, 12,000 rpm). The supernatant was recovered and total protein concentration was assessed by Bio-Rad assay. Total proteins were resolved on a 12% SDS-PAGE gel and transferred onto a nitrocellulose membrane. Primary antibodies; Anti-Goat H6PD (in house), Anti-Mouse NAMPT (Bethyl A300-372A), Anti-Rabbit NRK1 (in house [20]), Anti-Rabbit NRK2 (in house), Anti-Rabbit CHOP (Cell Signaling D46F1 5554), Total Lysine Ac (Cell Signaling 9441), Anti-Mouse Rodent OXPHOS Mitoprofile (Abcam ab110413), IDH2 K413 ac (GeneTel AC0004), IDH2 Total (Abcam ab55271), H3K9ac (Cell Signaling 9649), H3K56ac (Cell Signaling 4243), ERO1a (Cell Signaling 3264), PDI (Cell Signaling 3501), alpha-Tubulin (Santa Cruz B-7 sc-5286). Secondary antibodies (Dako) anti-mouse, anti-goat and anti-rabbit conjugated with HRP added at a dilution of 1/10,000. Equal loading of protein content was verified using alpha-tubulin as a housekeeping protein and bands visualised using ECL detection system (GE Healthcare, UK). Bands were measured using Image J densitometry, and normalised to those of loading control (alpha tubulin).

### High-resolution respirometry of permeabilised muscle fibres by Oroboros

Respirometry studies in tibialis anterior (TA) and soleus (SOL) skeletal muscle myofibres were performed using high-resolution respirometry (Oroboros Instruments, Innsbruck, Austria). Fresh skeletal muscle (5 mg) was stored in ice-cold 2mls BIOPS buffer. Any connective tissue and adipose was discarded and 20 μl of Saponin stock solution for permeabilisation of muscle fibres (5 mg Saponin per ml BIOPS buffer) added. The samples were then incubated for 30 min at 4 °C. Muscles were washed in Mir05 for 10 min at 4 °C before drying on filter paper. Approximately 3mgs of muscle tissue was introduced into the chamber and left for > 10 min to give a stable baseline signal, re-oxygenation occurred once O_2_ concentration reached 250 μM. Sequential addition of inhibitors and substrates occurred as per Oroboros Instruments specified protocol for fatty acid oxidation.

### NAD^+^ quantification by colorimetric assay

NAD^+^ was extracted from flash frozen skeletal muscle and NAD^+^/NADH quantified using the NAD^+^/NADH Assay kit (BioAssay Systems) according to the manufacturers instructions.

### Untargeted metabolomic measurement

Twenty milligrams of skeletal muscle previously flash frozen tissue from TA or SOL was placed in dry ice. Metabolites were extracted into 0.5 ml of ice-cold LC-MS grade methanol:acetonitrile:water (50:30:20). Samples were pulverised and stored at 4 °C for 10 min before centrifugation at 0 °C 1300 rpm for 15 min. Supernatant was then transferred to HPLC vials ready for LC-MS analysis. To preclude variables introduces from preparing samples separately all samples were prepared simultaneously in a randomised order and re-randomised before injection in the LC-MS. Untargeted metabolomics was carried out by using an Accela HPLC system interfaced to an Exactive Orbitrap mass spectrometer (Thermo Fisher Scientific, Bremen, Germany) was used for the liquid chromatographic separations. ZIC-pHILIC (150 × 4.6 mm, 5 μm) HPLC column supplied by HiChrom (Reading, UK) were used. Samples were run on LC-MS under the following conditions: the ZIC-pHILIC mobile phase consisted of 20 mM ammonium carbonate in HPLC-grade water (A) and acetonitrile (B); the solvent gradient used was 80% B (0 min), 20% (30 min), 8% (31–36 min), and 80% (37–45 min) at a flow rate of 0.3 mL/min. The nitrogen sheath and auxiliary gas flow rates were maintained at 50 and 17 arbitrary units. The electrospray ionisation (ESI) interface was employed in a positive/negative dual polarity mode, with a spray voltage of 4.5 kV for positive mode and 4.0 kV for negative mode, while the ion transfer capillary temperature was set at 275 °C. Full scan data were obtained in the mass-to-charge ratio (m/z) between 75 and 1200 amu for both ionisation modes. The data were collected and processed using Xcalibur 2.1.0 software (Thermo Fisher Scientific, Bremen, Germany). Data Extraction and Statistical Analysis The data were extracted using MZMatch software (SourceForge, La Jolla, USA). A macro-enabled Excel Ideom file was used to filter, compare and identify the metabolites. The metabolite lists obtained from these searches were then carefully evaluated manually by considering the quality of their peaks and the metabolites were matched with the retention times of authentic standards mixtures run in the same sequences. Library searches were also used for identification and carried out against accurate mass data of the metabolites in the Human Metabolome Data Base and KEGG (Kyoto Encyclopedia of Genes and Genomes). All metabolites were within 3 ppm of their exact masses. Univariate comparisons using paired t-tests were performed using Microsoft Excel for performing paired t-tests and differences were considered significant at *p* < 0.05.

### Targeted metabolomics experiment

Skeletal muscle (quadriceps), liver and plasma were collected from WT and H6PDKO littermates, frozen immediately and stored until analysis at − 80 °C. Targeted metabolomics measurements were performed in the Metabolomics Platform of the Genome Analysis Center of the Helmholtz Zentrum München. Frozen tissue was homogenized using homogenization tubes containing ceramic beads (1.4 mm diameter) and a Precellys 24 homogenizer with an integrated cooling unit. To each mg of frozen liver or muscle tissue 3 μL of a dry ice cooled mixture of ethanol/phosphate buffer (85/15 v/v) were added. Samples were homogenized at − 4 °C for three times over 20 s at 5500 rpm with 30 s pause intervals to ensure constant temperatures during homogenization. After homogenization, the samples were centrifuged at 4 °C and 2300×g for 5 min. Either 10 μL of the tissue supernatant or 10 μL plasma were used for the assay. Metabolite quantification was based on FIA-ESI-MS/MS (flow injection-electrospray ionisation-triple quadrupole mass spectrometry) and the Absolute*IDQ*™ p150 kit (BIOCRATES Life Sciences AG, Innsbruck, Austria). A detailed description of the tissue preparation and the p150 assay have been published [21]. Sample handling was performed by a Hamilton Microlab STARTM robot (Hamilton Bonaduz AG, Bonaduz, Switzerland) and a Ultravap nitrogen evaporator (Porvair Sciences, Leatherhead, U.K.), beside standard laboratory equipment. Mass spectrometric analyses were done on an API 4000 triple quadrupole system (Sciex Deutschland GmbH, Darmstadt, Germany) equipped with a 1200 Series HPLC (Agilent Technologies Deutschland GmbH, Böblingen, Germany) and a HTC PAL auto sampler (CTC Analytics, Zwingen, Switzerland) controlled by the software Analyst 1.5. Compound identification and quantification were based on multiple reaction monitoring measurements (MRM) and appropriate internal standards. The concentrations of plasma and tissue homogenate were reported in μM. In total, 163 Metabolites were quantified but only acylcarnitines were included in the pathway analyses due to phenotype assignment.

### Metabolomic set enrichment analysis

We conducted metabolite set enrichment analysis using MetaboAnalyst [22, 23]. Significantly dysregulated metabolites from the unbiased measurement in H6PDKO compared to WT TA muscle were uploaded to Metaboanalyst. These were processed with the over-representation analysis (ORA) algorithm and the metabolic pathway associated metabolite library set. This examines the group of metabolites using Fisher’s exact test, calculating the probability of seeing a particular number of metabolites containing the biological pathway of interest in the list of metabolites.

### Statistical analysis

Students T-test or ANOVA statistical comparisons were used with the Graphpad Software Inc. Prism version 5. Data presented as mean ± SEM with statistical significance determined as *. *p* < 0.05, **. *p* < 0.01, ***. *p* < 0.001. Unpaired T-Test compared treatments or genotypes. Statistical analysis derived from real-time PCR data was determined using dCt values throughout.

## Results

### Alterations in nicotinamide metabolism in H6PDKO muscle

**Fig. 1:**
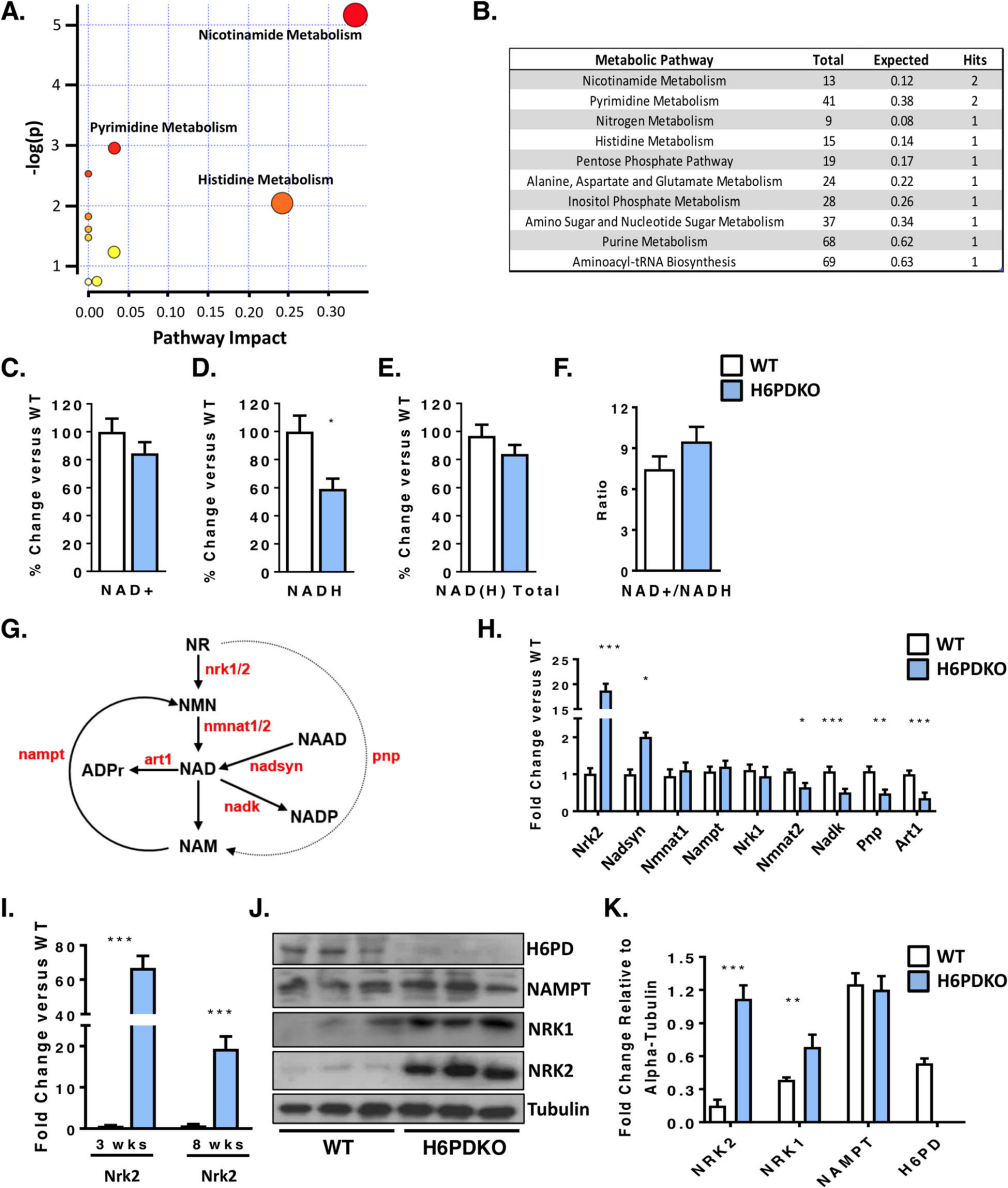
Altered NAD^+^ salvage pathway in H6PDKO muscle. **a** Pathway analysis of unbiased metabolomics from WT (*n* = 3) and H6PDKO (*n* = 3) tibialis anterior (TA) skeletal muscle. **b** Numerical output from pathway analysis of WT and H6PD KO tibialis anterior skeletal muscle. Values shown from pathway analysis. **c**-**f** Quantification of NAD^+^, NADH, total NAD(H) and ratio of NAD^+^/NADH in WT (*n* = 7) and H6PDKO (*n* = 6) TA muscle. **g** Schematic representation of the biosynthetic generation of NAD from nicotinamide Riboside (NR) and nicotinamide (NAM) salvage, enzymes shown in red. **h** qRT-PCR of NAD^+^ synthesis and salvage genes in WT (*n* = 7) and H6PDKO (*n* = 7) TA. **i** Expression of NRK2 transcript in TA muscle of WT and H6PDKO mice at 3 and 8 weeks of age. **j**-**k** Western blotting and quantification of WT (*n* = 9) and H6PDKO (*n* = 9) TA lysates. *P < 0.05, **P < 0.01 and ***P < 0.001

Global and skeletal-muscle specific knockout of H6PD provokes a myopathy characterised by metabolic stress, abnormal SR structure, SR stress and activation of the unfolded protein response. We subjected Tibialis Anterior (TA) skeletal muscle from WT and H6PDKO mice to an unbiased metabolomic screen performed by LC-MS to better understand the role H6PD plays in muscle cell metabolism and SR redox maintenance. Those metabolites significantly dysregulated in H6PDKO TA were subject to an overrepresentation analysis algorithm comparing probability of occurrence against pathway associated libraries using MetaboAnalyst [22, 24]. The most over represented pathways were those involved in nicotinamide and pyrimidine metabolism (Fig. 1a-b). Nicotinamide (NAM) generation is essential to the salvage and biosynthesis of both NAD^+^ and NADH. Therefore, changes in NAM metabolism may indicate shifts in skeletal muscle NAD^+^ availability as a result of perturbed SR NAD(P)(H) due to H6PD deficiency. In support of this specific metabolite changes with potential relevance to SR redox included elevated trimethyl-L-Lysine; a constituent of nuclear histone proteins and a precursor to carnitine and fatty acid oxidation in the mitochondria, elevated 3-Hydroxysebasicacid; associated with peroxisomal disorders and glycogen storage disease [25, 26] suggestive of a defect in fatty acids synthesis and metabolic defects such as medium chain acyl-CoA dehydrogenase deficiency, and finally elevated Cis-5-Tetradecenoylcarnitine; a marker of altered beta-oxidation contributing to skeletal myopathy [27].

We measured NAD^+^/NADH levels and while no significant change was noted in NAD^+^, there was a significant 40% reduction in NADH in H6PDKO mice (Fig. 1c-f). Maintenance of cellular NAD^+^ pools through utilisation of multiple salvage and biosynthetic enzymes is critical for NAD(P)^+^/NAD(P)H homeostasis (schematic Fig. 1g). We therefore examined expression of the enzymes involved in these pathways and observed changes in the mRNAs of genes determining the NAD^+^ associated metabolome (Fig. 1h). The most prominent adaptation was an increase in the skeletal muscle-restricted *nicotinamide riboside kinase 2* (Nrk2) gene, whilst the constitutively expressed salvage enzymes Nrk1 and Nampt were unchanged. Responsible for the phosphorylation of the NAD^+^ precursor nicotinamide riboside (NR) into nicotinamide mononucleotide (NMN), Nrk2 has previously been shown to be elevated in models of muscle energy stress and cardiomyopathy [28]. Downregulation of NAD kinase may limit generation of NADP^+^, and may indicate a response to prevent NAD(H). Purine Nucleoside Phosphorylase (Pnp) (which converts NR to NAM) and the NAD^+^ utilising ADP-ribosyltransferase (Art1) were both downregulated, which may also reflect a response to maintain NAD(H). We further evaluated the expression of NAD^+^ salvage genes prior to phenotypic presentation of myopathy in 3 week old mice. At this age *Nrk2* was the only changed transcript, being upregulated > 20-fold, suggesting this is a primary adaptive metabolic response to H6PD deficiency (Fig. 1i). Western blotting confirmed elevation of NRK2 at the protein level and interestingly also suggested upregulation of NRK1 protein, while expression of the rate-limiting NAMPT NAD^+^ salvage pathway remained unchanged (Fig. 1j-k).

### H6PDKO skeletal muscle has reduced mitochondrial fatty acid oxidative capacity and widespread changes in acylcarnitines

**Fig. 2:**
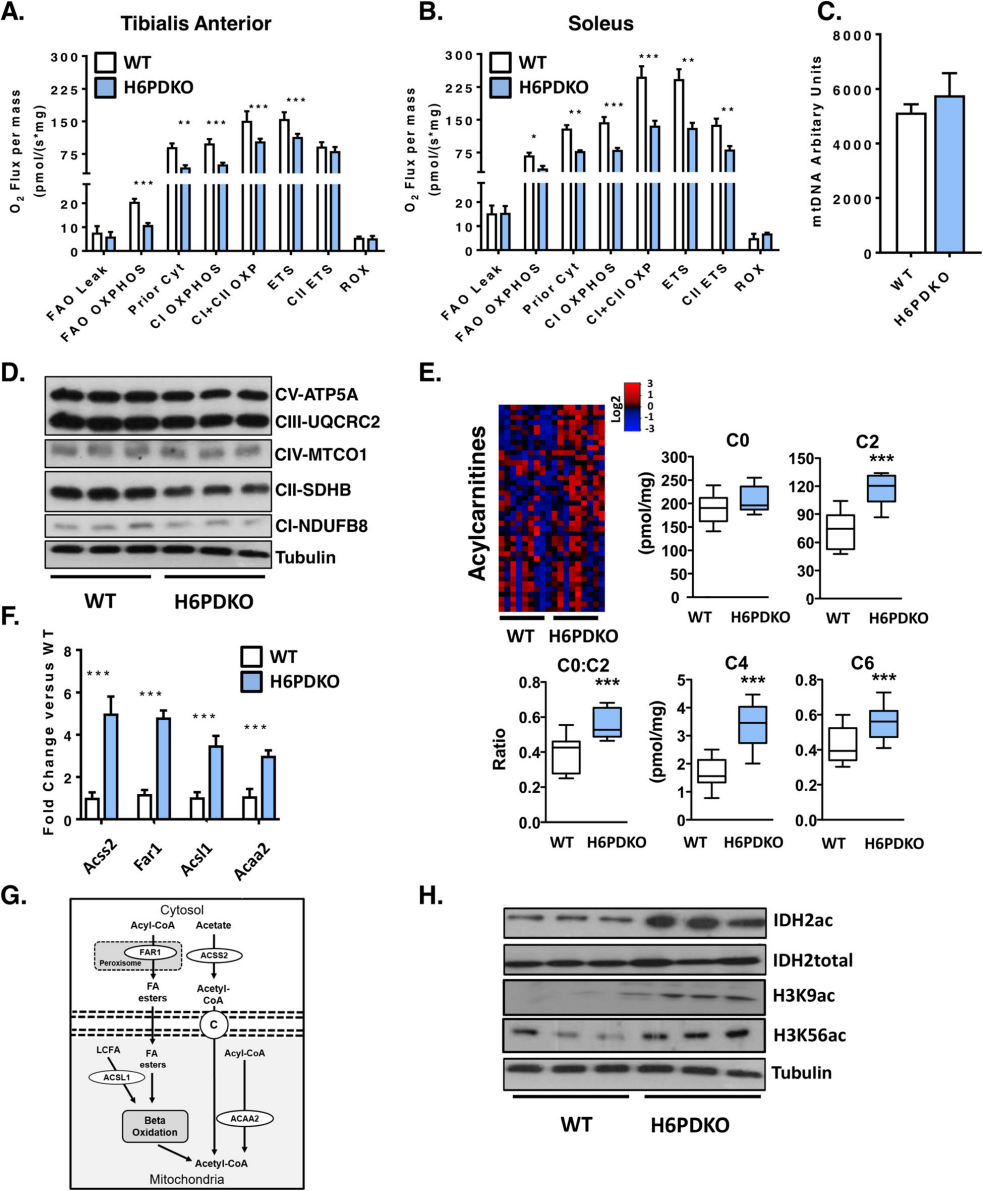
Impaired mitochondrial fatty acid oxidation in H6PDKO skeletal muscle. **a** High resolution respirometry of fatty acid oxidation in permeabilised tibialis anterior WT (*n* = 3) in and H6PDKO (*n* = 3). **b** High-resolution respirometry of fatty acid oxidation using WT (*n* = 3) and H6PDKO (*n* = 3) permeabilised. **c** Mitochondrial DNA (mtDNA) quantification of WT (*n* = 7) and H6PDKO (*n* = 7) muscle, measured using qRT-PCR. **d** Western blots of WT and H6PDKO protein lysates (*n* = 9) probed for oxidative phosphorylation enzyme subunit abundance. **e** Acylcarnitine species levels in WT (*n* = 9) and H6PDKO (*n* = 11) muscle measured using GC-MS/MS. Data expressed as heat-maps with log2 values representing metabolite abundance in WT and H6PDKO. Box and Whisker plots showing significantly altered short acylcarnitines. **f** qRT-PCR measurement of genes critical carnitine and fatty acids metabolism in WT (*n* = 7) and H6PDKO (*n* = 7) TA. **g** Schematic showing carnitine and fatty acid metabolism between cytosol and mitochondria. **h** Western blots of acetylated proteins within WT (*n* = 6) and H6PDKO (*n* = 6) skeletal muscle. *P < 0.05, **P < 0.01 and ***P < 0.001

Changes in NAD^+^/NADH turnover and availability can impact mitochondrial function [29–31]. We therefore investigated this in permeabilised skeletal muscle fibres from H6PDKO TA and SOL muscle using high-resolution mitochondrial respirometry. Both TA and SOL muscle have impaired oxygen consumption when exposed to L-Octanoylcarnitine as an energetic substrate, indicating a decreased ability to utilise substrates for fatty acid beta-oxidation and overall respiratory capacity (Fig. 2a, b). This defect was more apparent in SOL muscle, likely representing its greater mitochondrial density (Fig. 2b). To understand if these measurements were a result of mitochondrial abundance we examined mtDNA and mitochondrial respiratory complex subunit abundance in WT and H6PDKO TA and found no differences suggesting that the defects in respiratory capacity were through impaired mitochondrial function (Fig. 2c-d).

To further examine the basis for dysfunctional lipid metabolism and impaired mitochondrial fatty acid oxidation we conducted a targeted lipidomic analysis of serum, liver and quadriceps from H6PDKO and WT mice (Additional file 1: Figure S1). Liver and serum from H6PDKO showed no significant changes in lipid profile. However, a striking effect for elevated short-chain acylcarnitine (C2, C3, C4, C5, C6 & C10:1) abundance in H6PDKO muscle tissue was seen. While free carnitine was not significantly different, the ratio of acetyl carnitine to free carnitine (C2:C0) was increased, possibly signifying an increased production rate or block in use of acetyl-CoA in H6PDKO muscle. (Fig. 2e).

Using whole genome expression array data, validated with real-time PCR, we also found that H6PDKO muscle have significant elevations in genes transcripts Acss2, Far1, Acsl1, and Acaa2, which converge on acylcarnitine and acetyl-CoA metabolism (Fig. 2f, g) [4]. Acetyl-CoA produced during fatty acid oxidation is important to the levels of histone acetylation and the regulation of gene expression [32, 33]. Accumulation of mitochondrial acetyl-CoA can lead to non-enzymatic acetylation of multiple proteins associated with energy metabolism [34, 35]. Protein lysates from H6PDKO muscle show increases in mitochondrial- Isocitrate Dehydrogenase 2 (IDH2) acetylation and nuclear histone 3 lysine 9 and lysine 56 acetylation, supporting the observation of dysfunctional fatty acid metabolism (Fig. 2h).

### Rescuing NAD^+^ metabolism using nicotinamide riboside

**Fig. 3:**
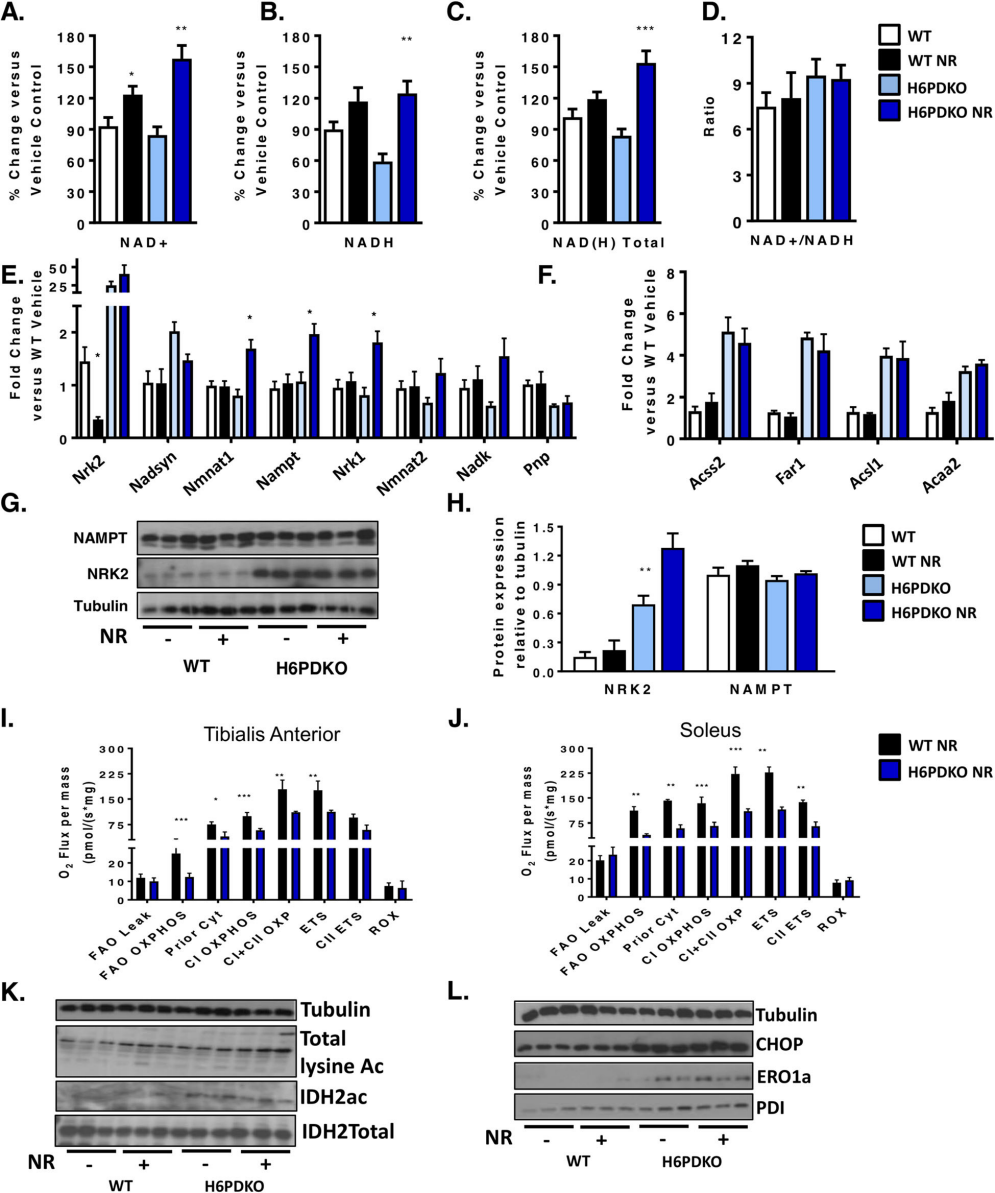
NR supplementation, NAD^+^ salvage and mitochondrial function in H6PDKO muscle. **a**-**d** NAD^+^, NAD(H), total NAD(H) quantification and ratio of NAD^+^/NADH in TA of WT and H6PDKO +/− intraperitoneal (i.p.) Nicotinamide Riboside (NR) (*n* = 6-9). **e** qRT-PCR data of genes involved in the biosynthesis of NAD^+^ and salvage of NAM in WT and H6PDKO muscle +/− i.p. NR (*n* = 6-9). **f** qRT-PCR data of genes critical carnitine and fatty acids metabolism in WT and H6PDKO muscle +/− i.p. NR (*n* = 6-9). **g**-**h** Western blots and quantification of the NAM salvage protein NAMPT and the skeletal muscle specific protein NRK2 (*n* = 6). **i** High-resolution respirometry of fatty acid oxidation in permeabilised TA tissue from WT and H6PDKO i.p NR (*n* = 3). **j** High-resolution respiration for fatty acid oxidation using WT and H6PDKO after i.p NR permeabilised SOL (*n* = 3). **k** Western blots showing total lysine acetylation and IDH2 acetylation in WT and H6PDKO muscle +/− i.p NR. **l** Western blots showing ER stress regulator CHOP and protein folding factors PDI and ERO1a in WT and H6PDKO +/− i.p NR. (*n* = 6-9). *P < 0.05, **P < 0.01 and ***P < 0.001

Changes in NAD(H) homeostasis in the absence of H6PD are associated with dysfunctional mitochondrial fatty acid oxidation and altered protein acetylation which is associated with an adverse metabolic phenotype (Fig. 2) [36]. In H6PDKO muscle NAD^+^ levels are not significantly lower, whereas NADH levels are 60% those of WT. It is recognised that upregulation of the NRK pathway is a response to metabolic stress in which there is a drain on NAD(H) homeostasis, requiring greater input through NR salvage [20, 28, 37]. Taking advantage of NRK2 upregulation we attempted to enhance NAD(H) availability through NR supplementation, which has extensively been shown as a means to augment metabolic resilience [20, 28, 38, 39]. We also asked if increasing NAD^+^ availability will reverse protein acetylation via NAD-dependent protein deacetylases [40–43]. We supplemented mice with NR (IP, 400 mg/kg twice daily over 4 consecutive days). Quantification of NAD(H) levels in the skeletal muscle of mice receiving NR showed it to rescue NAD(H)levels (Fig. 3a-d). Furthermore, the ability to elevate NAD(H) was more pronounced in H6PDKO muscle with upregulated NRK pathway activity.

We observed that after NR supplementation NRK2 expression was decreased in WT but not H6PDKO muscle. However, NR did lead to increased expression of NMNAT1, NAMPT and NRK1 in H6PDKO muscle, which was not observed in WT (Fig. 3e). NAD^+^ boosting did not suppress upregulation of Acyl-CoA and lipid metabolism gene expression found in H6PDKO mice (Fig. 3f). Protein quantification of NAMPT and NRK2 showed no change in abundance (Fig. 3g-h). Following NR supplementation we again performed high-resolution mitochondrial respirometry of fatty acid oxidation and found no improvement in mitochondrial O2 flux in NR supplemented WT or H6PDKO muscle (Fig. 3i-j).

To further assess the effects of augmented NAD(H) availability we examined protein acetylation in response to NR. Global and IDH2 acetylation were maintained at untreated H6PDKO levels (Fig. 3k). Finally, as the H6PDKO muscle demonstrated extensive activation of the unfolded protein response, potentially a consequence of altered SR NADPH/NADP^+^ ratio and impaired protein folding [2, 44, 45] we examined UPR markers CHOP and the protein folding regulators ERO1a and PDI levels and showed them to remain elevated in H6PDKO mice despite NR supplementation and increased NAD(H) availability (Fig. 3l). These data imply that perturbed SR NAD(P)(H) as a function of H6PD deficiency cannot be overcome by boosting NAD(H) availability according to this study design.

### NRK2 is dispensable in H6PDKO myopathy

**Fig. 4:**
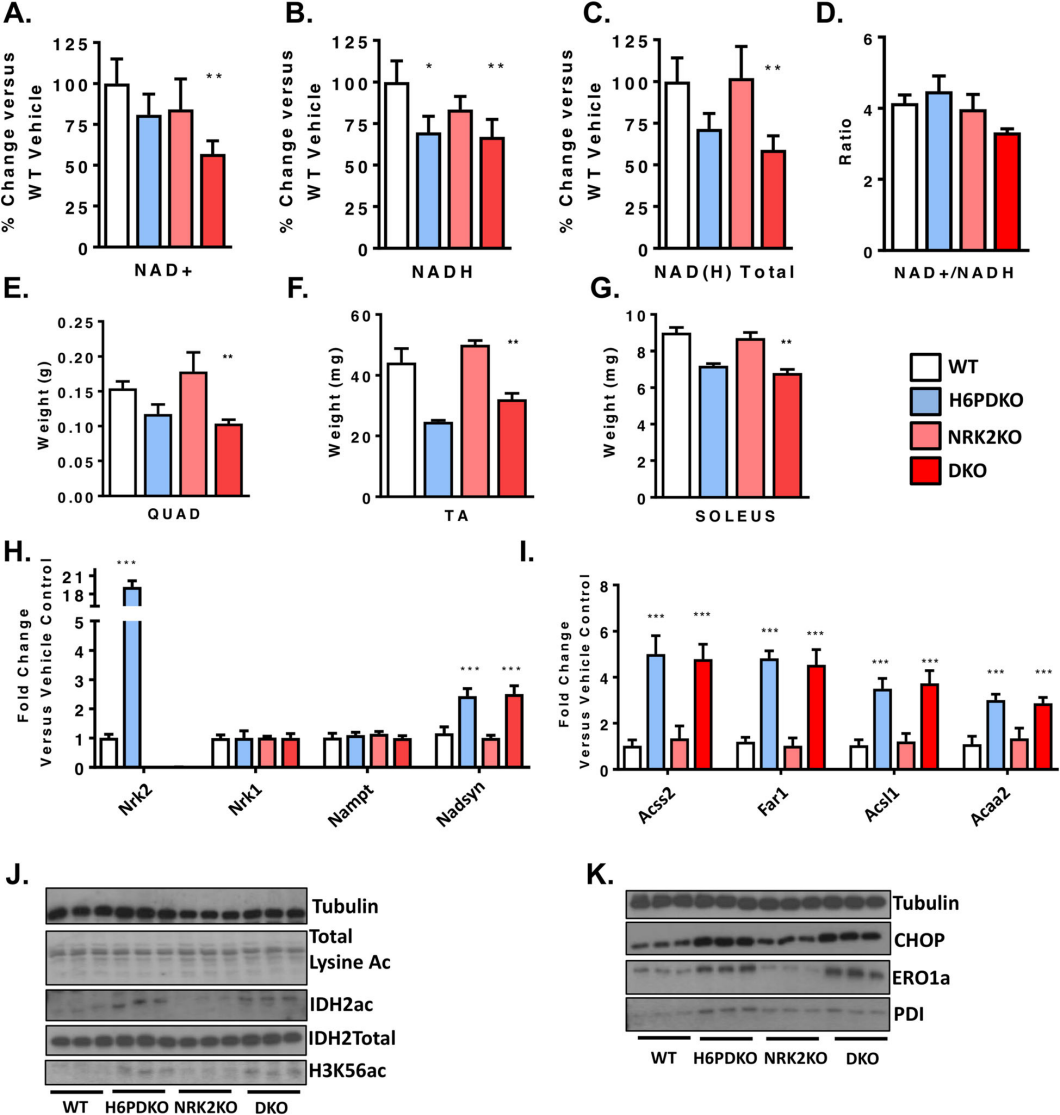
Molecular and phenotypic analysis of H6PD/NRK2 Double knockout mice. **a**-**d** NAD^+^, NADH, total NAD(H) and ratio of NAD^+^/NADH in WT, H6PDKO, NRK2KO and DKO (H6PDKO-NRK2KO) TA muscle (*n* = 3-6). **e**-**g** Skeletal muscle tissue weights from WT, H6PDKO, NRK2KO and H6-NRK2 Double Knockout (DKO) (*n* = 3-6). **h** qRT-PCR of NAD^+^ biosynthetic gene expression in WT, H6PDKO, NRK2 KO and DKO in TA (*n* = 3-6). **i** qRT-PCR of mitochondrial and acyl-CoA genes in TA of WT, H6PDKO, NRK2 KO and DKO (*n* = 3-6). **j** Western blots of total lysine acetylation, IDH2 and H3K56 acetylation in WT, H6PDKO, NRK2KO and DKO muscle protein lysates (*n* = 3-6). **k** Western blots of CHOP and protein folding factors PDI and ERO1a in WT, H6PDKO, NRK2 KO and DKO muscle lysates (*n* = 3-6). *P < 0.05, **P < 0.01 and ***P < 0.001

We also investigated the role of the NRK2 pathway in defending muscle function in the absence of H6PD using double knockout (DKO) mice. We reasoned DKO mice would have an exacerbated myopathy, and may even reduce survival. However, DKO mice were viable, survived at the anticipated frequency and generally indistinguishable from H6PDKO mice. Quantification of NAD^+^ levels revealed that DKO TA has significantly decreased NAD^+^ levels compared to WT and to a greater degree than in H6PD deficiency alone, with NADH and total NAD(H) remaining depressed at H6PDKO levels (Fig. 4a-d). The increased expression of NRK2 would therefore appear to be able to defend aspects of the NAD metabolome in H6PD deficiency. NRK2 deficiency alone had minimal impact in WT mice as previously reported, and likely due to compensatory activity of the NRK1 enzyme [20]. DKO skeletal muscle exhibited myopathy and atrophy to an equivalent degree as in H6PDKO (Fig. 4e-g). Expression of genes for NAD^+^ biosynthesis and Acyl-CoA metabolism revealed no significant differences in the DKO muscle compared to H6PDKO, with NRK2KO displaying no change compared to WT (Fig. 4h, i). Finally we examined protein acetylation and ER stress and UPR markers, all of which were, again, unchanged from the levels seen in H6PDKO muscle (Fig. 4j, k).

## Discussion

In this work we show that upregulation of NRK2 mediated salvage of NR into NAD^+^ is an early adaptation to perturbed muscle SR NAD(P)(H) homeostasis and impaired mitochondrial energy production in H6PD deficiency. This study demonstrates that NR supplementation can defend NAD(H) levels within H6PD deficient muscle, but this, and ablation of the stress responsive NRK2 pathway have little impact to limit or worsen myopathy.

The nicotinamide riboside kinases 1 and 2 (NRKs1/2) form part of the salvage pathway that converts NR into NMN through to NAD^+^ [20]. Importantly, extracellular NMN conversion to NAD^+^ is NRK dependent, requiring it to be dephosphorylated to NR to be utilised as an NAD^+^ precursor [37]. NRK1 is thought to be the dominant and rate-limiting source of NR phosphorylation [20, 37] in most tissues, with NRK2 expression being limited to skeletal and cardiac muscle tissues [37]. Upregulation of NRK2 has been observed as a primary response to metabolic energy stress associated with depletion of NAD^+^ in genetic models of cardiomyopathy and in a model of post-injury skeletal muscle repair [20, 28, 46]. Despite its limited expression in non-muscle tissues, NRK2 has also been shown to be induced in axonal neurons in response to damage, further supporting a role in response to cellular damage or metabolic stress [47].

Supplementation of the vitamin B3 NR as an NAD^+^ precursor is well tolerated in both mice and humans and effective in elevating muscle NAD^+^ levels and alleviating aspects of the pathology seen in muscular dystrophy, mitochondrial myopathy and ageing [48–50]. Indeed, NR supplementation has proven to be effective in alleviating cardiac defects associated with genetic cardiomyopathies’ [28, 46]. We report that NR was successful at enhancing NAD(H) levels in H6PDKO muscle but unable to rescue physiological or molecular defects associated with the metabolic myopathy, despite elevated NRK2 activity. It is reasonable to suggest more sustained NR delivery, beyond the 4 day protocol used here, may have revealed effects.

We also show that abolishing NRK2 upregulation in H6PDKO muscle confers no discernible disadvantage, with DKO mice indistinguishable from H6PDKO mice, despite further deterioration in NAD^+^ availability. We have previously shown that NRK1 is able to compensate for NRK2 deficiency in terms of NAD^+^ salvage, and as NRK1 protein is also upregulated in H6PDKO muscle, may again do so in this context [20]. Also, muscle-specific NAMPT KO mice are viable and only develop myopathy after several months despite NAD^+^ levels at 90-95% those of WT mice [31]. We were unable to ascertain the levels of NR available to the NRK2 pathway in H6PD deficiency, and suggest that despite NRK2 elevation, NR availability will be limiting, even with supplementation [51].

Upregulation of NRK2 may be an early adaptive response to metabolic stress and the need to defend NAD^+^ availability. In cardiomyocytes the NRK2 gene is activated by energy stress and NAD^+^ depletion through AMP-activated protein kinase and peroxisome proliferator-activated receptor α dependant mechanisms [28, 52]. This may be important in exercise adaptation as aged NRK2 KO mice show maladaptive metabolic responses to exercise in cardiac and skeletal muscle [46]. We therefore generated H6PD/NRK2 double knockout mice to examine this further. However, loss of the stress responsive NRK2 pathway had little impact to limit or worsen H6PD-driven myopathy, despite further deterioration in NAD(H) availability. Indeed this worsening of NAD^+^ availability demonstrates the importance of the endogenous pool of NR available to defend against situations of metabolic stress induce NAD(H) depletion.

The role NRK2 may play as a direct response to perturbed SR NAD(P)(H) homeostasis remains unclear given NAD^+^ precursor supplementation had no impact on acetlycarnitine metabolism or mitochondrial function. It is possible that elevated NRK2 is a direct response to perturbed SR NAD(P)(H) homeostasis and regulated through a mechanism distinct from that initiated upon energy stress and NAD^+^ depletion. This notion may support the concept of SR sensing and exchange of nucleotides, nucleosides or other precursors involved in NAD(P)(H) homeostasis. Recent findings have identified transporters able to carry NAD^+^ and NMN across biological membranes. Importantly, NAD^+^ has been shown to be transported into the mitochondria and as such increases the potential for other compartments, such as the ER/SR, to transport NAD^+^ and associated precursors [53]. Indeed, Slc12a8 has recently been identified as an NMN transporter regulating intestinal NAD^+^ metabolism [54]. Although an NR transporter has been identified in yeast, there remains no conclusive evidence for a dedicated NR transporter in mammalian cells [55].

## Conclusion

These data identify activation of the NRK2 pathway, and concurrent changes in NAD^+^ metabolism, as an early response to perturbations in ER/SR NAD(P)(H) homeostasis as a result of H6PD deficiency in skeletal muscle. Whether upregulation of NRK2 mediated NAD(P)(H) salvage is a response to mitochondrial dysfunction and energy stress, or directly to perturbation in NADP(H) availability remains to be determined. While H6PD deficiency results in a deteriorating and complex metabolic and structural myopathy that cannot be rescued with NAD^+^ supplementation, it remains an intriguing model linking muscle SR NAD(P)(H) mediated redox and energy metabolism.

## Supplementary information

**Additional file 1: Figure S1.** Acylcarnitine levels of WT and H6PDKO in skeletal muscle, serum and liver. Metabolite signals are presented as Log2 signal intensity.

**Additional file 2: Figure S2.** NRK2 detection with Western blot. (a) Western Blot of skeletal muscle lysates taken from TA muscle of Cre-negative NRK2 (equivalent to WT), skeletal muscle specific NRK2 overexpressing mice (NRK2^ActaCre+/+^), Wild type (WT) and NRK2 knockout (KO). NRK2 protein is evident at 22 kDa in all WT and transgenic lysates. It is absent in NRK2 KO tissue. (b) Western Blot of skeletal muscle lysates taken from WT(F4 & F5) and H6PD KO(F1 & F2) mice. Alongside are muscle lysates form WT and NRK2 knockout mice. The left panel shows blots incubated with the NRK2 antibody and the blocking peptide and results in no signal for NRK2 being obtained. Panel on the right shows blots incubated with NRK2 antibody, demonstrating bands at 22 kDa.

## Data Availability

Datasets used in this study are available from the author upon request.

## Abbreviations

ER/SR: Endoplasmic/Sarcoplasmic Reticulum
H6PD: Hexose-6-Phosphate Dehydrogenase
NAD^+^: Nicotinamide Adenine Dinucleotide
NMN: Nicotinamide Mononucleotide
NR: Nicotinamide Riboside
NRK: Nicotinamide Riboside Kinase
SOL: Soleus
TA: Tibialis Anterior

## References

[1] Rogoff D (2007). Abnormalities of glucose homeostasis and the hypothalamic-pituitary-adrenal axis in mice lacking hexose-6-phosphate dehydrogenase. Endocrinology.

[2] Rogoff D, Black K, McMillan DR, White PC (2010). Contribution of hexose-6-phosphate dehydrogenase to NADPH content and redox environment in the endoplasmic reticulum. Redox Rep.

[3] Lavery GG (2006). Hexose-6-phosphate dehydrogenase knock-out mice lack 11 beta-hydroxysteroid dehydrogenase type 1-mediated glucocorticoid generation. J Biol Chem.

[4] Lavery GG (2008). Deletion of hexose-6-phosphate dehydrogenase activates the unfolded protein response pathway and induces skeletal myopathy. J Biol Chem.

[5] White PC, Rogoff D, McMillan DR (2008). Physiological roles of 11 beta-hydroxysteroid dehydrogenase type 1 and hexose-6-phosphate dehydrogenase. Curr Opin Pediatr.

[6] Zielinska AE, Walker EA, Stewart PM, Lavery GG (2011). Biochemistry and physiology of hexose-6-phosphate knockout mice. Mol Cell Endocrinol.

[7] Atanasov AG, Nashev LG, Schweizer RA, Frick C, Odermatt A (2004). Hexose-6-phosphate dehydrogenase determines the reaction direction of 11beta-hydroxysteroid dehydrogenase type 1 as an oxoreductase. FEBS Lett.

[8] Draper N (2003). Mutations in the genes encoding 11beta-hydroxysteroid dehydrogenase type 1 and hexose-6-phosphate dehydrogenase interact to cause cortisone reductase deficiency. Nat Genet.

[9] White PC, Rogoff D, McMillan DR, Lavery GG (2007). Hexose 6-phosphate dehydrogenase (H6PD) and corticosteroid metabolism. Mol Cell Endocrinol.

[10] Lavery GG (2013). Novel H6PDH mutations in two girls with premature adrenarche: 'apparent' and 'true' CRD can be differentiated by urinary steroid profiling. Eur J Endocrinol.

[11] Bujalska IJ (2008). Lack of hexose-6-phosphate dehydrogenase impairs lipid mobilization from mouse adipose tissue. Endocrinology.

[12] Lavery GG (2012). Lack of significant metabolic abnormalities in mice with liver-specific disruption of 11beta-hydroxysteroid dehydrogenase type 1. Endocrinology.

[13] Semjonous NM (2011). Hexose-6-phosphate dehydrogenase contributes to skeletal muscle homeostasis independent of 11beta-hydroxysteroid dehydrogenase type 1. Endocrinology.

[14] Foster CA, Mick GJ, Wang X, McCormick K (2013). Evidence that adrenal hexose-6-phosphate dehydrogenase can effect microsomal P450 cytochrome steroidogenic enzymes. Biochim Biophys Acta.

[15] Love NR (2015). NAD kinase controls animal NADP biosynthesis and is modulated via evolutionarily divergent calmodulin-dependent mechanisms. Proc Natl Acad Sci U S A.

[16] Garten A (2015). Physiological and pathophysiological roles of NAMPT and NAD metabolism. Nat Rev Endocrinol.

[17] Canto C, Sauve AA, Bai P (2013). Crosstalk between poly(ADP-ribose) polymerase and sirtuin enzymes. Mol Asp Med.

[18] White AT, Schenk S (2012). NAD(+)/NADH and skeletal muscle mitochondrial adaptations to exercise. Am J Physiol Endocrinol Metab.

[19] Zielinska AE, Fletcher RS, Sherlock M, Doig CL, Lavery GG (2017). Cellular and genetic models of H6PDH and 11beta-HSD1 function in skeletal muscle. Cell Biochem Funct.

[20] Fletcher RS (2017). Nicotinamide riboside kinases display redundancy in mediating nicotinamide mononucleotide and nicotinamide riboside metabolism in skeletal muscle cells. Mol Metab.

[21] Römisch-Margl W, Prehn C, Bogumil R, Röhring C, Suhre K, Adamski J (2011). Procedure for tissue sample preparation and metabolite extraction for high-throughput targeted metabolomics. Metabolomics.

[22] Xia J, Sinelnikov IV, Han B, Wishart DS (2015). MetaboAnalyst 3.0--making metabolomics more meaningful. Nucleic Acids Res.

[23] Xia J, Wishart DS (2011). Web-based inference of biological patterns, functions and pathways from metabolomic data using MetaboAnalyst. Nat Protoc.

[24] Xia J, Mandal R, Sinelnikov IV, Broadhurst D, Wishart DS (2012). MetaboAnalyst 2.0--a comprehensive server for metabolomic data analysis. Nucleic Acids Res.

[25] Tserng KY, Jin SJ, Kerr DS, Hoppel CL (1991). Urinary 3-hydroxydicarboxylic acids in pathophysiology of metabolic disorders with dicarboxylic aciduria. Metabolism.

[26] Bergoffen J, Kaplan P, Hale DE, Bennett MJ, Berry GT (1993). Marked elevation of urinary 3-hydroxydecanedioic acid in a malnourished infant with glycogen storage disease, mimicking long-chain L-3-hydroxyacyl-CoA dehydrogenase deficiency. J Inherit Metab Dis.

[27] Strauss AW (1995). Molecular basis of human mitochondrial very-long-chain acyl-CoA dehydrogenase deficiency causing cardiomyopathy and sudden death in childhood. Proc Natl Acad Sci U S A.

[28] Diguet N (2018). Nicotinamide Riboside Preserves Cardiac Function in a Mouse Model of Dilated Cardiomyopathy. Circulation.

[29] Agerholm M (2018). Perturbations of NAD(+) salvage systems impact mitochondrial function and energy homeostasis in mouse myoblasts and intact skeletal muscle. Am J Physiol Endocrinol Metab.

[30] Uddin GM, Youngson NA, Sinclair DA, Morris MJ (2016). Head to head comparison of short-term treatment with the NAD(+) precursor nicotinamide mononucleotide (NMN) and 6 weeks of exercise in obese female mice. Front Pharmacol.

[31] Frederick DW (2016). Loss of NAD homeostasis leads to progressive and reversible degeneration of skeletal muscle. Cell Metab.

[32] Pougovkina O (2014). Mitochondrial protein acetylation is driven by acetyl-CoA from fatty acid oxidation. Hum Mol Genet.

[33] Mews P (2017). Acetyl-CoA synthetase regulates histone acetylation and hippocampal memory. Nature.

[34] Lozoya Oswaldo A, Wang Tianyuan, Grenet Dagoberto, Wolfgang Taylor C, Sobhany Mack, Ganini da Silva Douglas, Riadi Gonzalo, Chandel Navdeep, Woychik Richard P, Santos Janine H (2019). Mitochondrial acetyl-CoA reversibly regulates locus-specific histone acetylation and gene expression. Life Science Alliance.

[35] Shi L, Tu BP (2015). Acetyl-CoA and the regulation of metabolism: mechanisms and consequences. Curr Opin Cell Biol.

[36] Muoio DM, Neufer PD (2012). Lipid-induced mitochondrial stress and insulin action in muscle. Cell Metab.

[37] Ratajczak J (2016). NRK1 controls nicotinamide mononucleotide and nicotinamide riboside metabolism in mammalian cells. Nat Commun.

[38] Trammell SA (2016). Nicotinamide riboside is uniquely and orally bioavailable in mice and humans. Nat Commun.

[39] Elhassan YS (2019). Nicotinamide Riboside Augments the Aged Human Skeletal Muscle NAD(+) Metabolome and Induces Transcriptomic and Anti-inflammatory Signatures. Cell Rep.

[40] Schwer B, Bunkenborg J, Verdin RO, Andersen JS, Verdin E (2006). Reversible lysine acetylation controls the activity of the mitochondrial enzyme acetyl-CoA synthetase 2. Proc Natl Acad Sci U S A.

[41] Hallows WC, Lee S, Denu JM (2006). Sirtuins deacetylate and activate mammalian acetyl-CoA synthetases. Proc Natl Acad Sci U S A.

[42] Jing E (2011). Sirtuin-3 (Sirt3) regulates skeletal muscle metabolism and insulin signaling via altered mitochondrial oxidation and reactive oxygen species production. Proc Natl Acad Sci U S A.

[43] Rardin MJ (2013). Label-free quantitative proteomics of the lysine acetylome in mitochondria identifies substrates of SIRT3 in metabolic pathways. Proc Natl Acad Sci U S A.

[44] Delaunay-Moisan A, Appenzeller-Herzog C (2015). The antioxidant machinery of the endoplasmic reticulum: protection and signaling. Free Radic Biol Med.

[45] Santos CX, Tanaka LY, Wosniak J, Laurindo FR (2009). Mechanisms and implications of reactive oxygen species generation during the unfolded protein response: roles of endoplasmic reticulum oxidoreductases, mitochondrial electron transport, and NADPH oxidase. Antioxid Redox Signal.

[46] Aguilar CA (2015). In vivo monitoring of transcriptional dynamics after lower-limb muscle injury enables quantitative classification of healing. Sci Rep.

[47] Sasaki Y, Araki T, Milbrandt J (2006). Stimulation of nicotinamide adenine dinucleotide biosynthetic pathways delays axonal degeneration after axotomy. J Neurosci.

[48] Martens CR (2018). Chronic nicotinamide riboside supplementation is well-tolerated and elevates NAD(+) in healthy middle-aged and older adults. Nat Commun.

[49] Khan NA (2014). Effective treatment of mitochondrial myopathy by nicotinamide riboside, a vitamin B3. EMBO Mol Med.

[50] Zhang H (2016). NAD(+) repletion improves mitochondrial and stem cell function and enhances life span in mice. Science.

[51] Liu L (2018). Quantitative Analysis of NAD Synthesis-Breakdown Fluxes. Cell Metab.

[52] Deloux R, Tannous C, Ferry A, Li Z, Mericskay M (2018). Aged nicotinamide riboside kinase 2 deficient mice present an altered response to endurance exercise training. Front Physiol.

[53] Davila A, et al. Nicotinamide adenine dinucleotide is transported into mammalian mitochondria. Elife. 2018;7. 10.7554/eLife.33246. 10.7554/eLife.33246 PMC6013257 29893687

[54] Grozio A (2019). Slc12a8 is a nicotinamide mononucleotide transporter. Nat Metab.

[55] Belenky PA, Moga TG, Brenner C (2008). Saccharomyces cerevisiae YOR071C encodes the high affinity nicotinamide riboside transporter Nrt1. J Biol Chem.

