# Supplementary figures and images for: Induction of the nicotinamide riboside kinase NAD+ salvage pathway in a model of sarcoplasmic reticulum dysfunction
Source: Skelet Muscle. 2020 Feb 19;10:5. doi: 10.1186/s13395-019-0216-z (PMC7031948; doi:10.1186/s13395-019-0216-z)

Supplementary Fig 1.

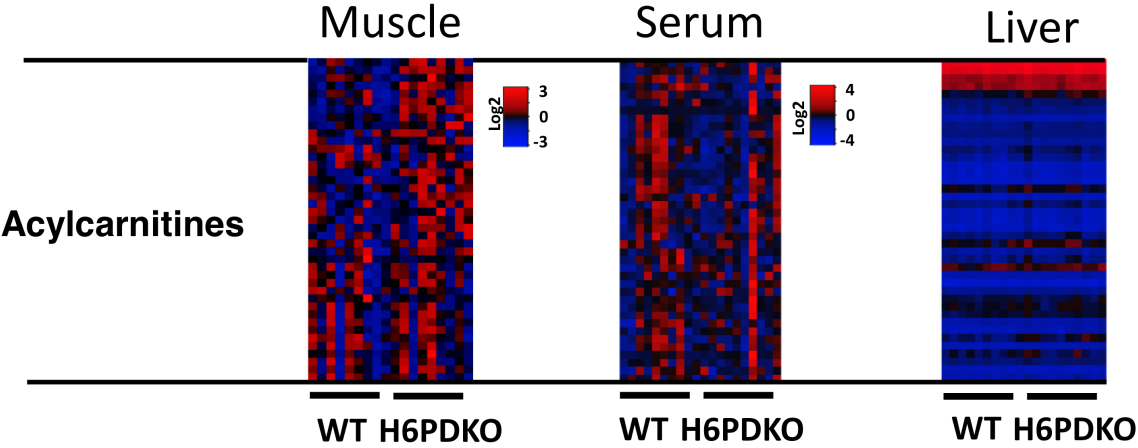

Supplement: Supplementary file 1 — Additional file 1: Figure S1. Acylcarnitine levels of WT and H6PDKO in skeletal muscle, serum and liver. Metabolite signals are presented as Log2 signal intensity. [file 13395_2019_216_MOESM1_ESM.pdf]

Supplementary Fig 2.

A.

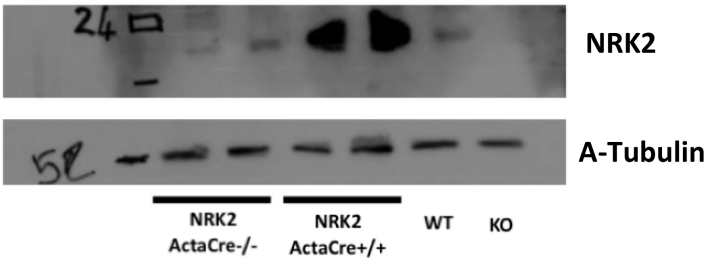

B.

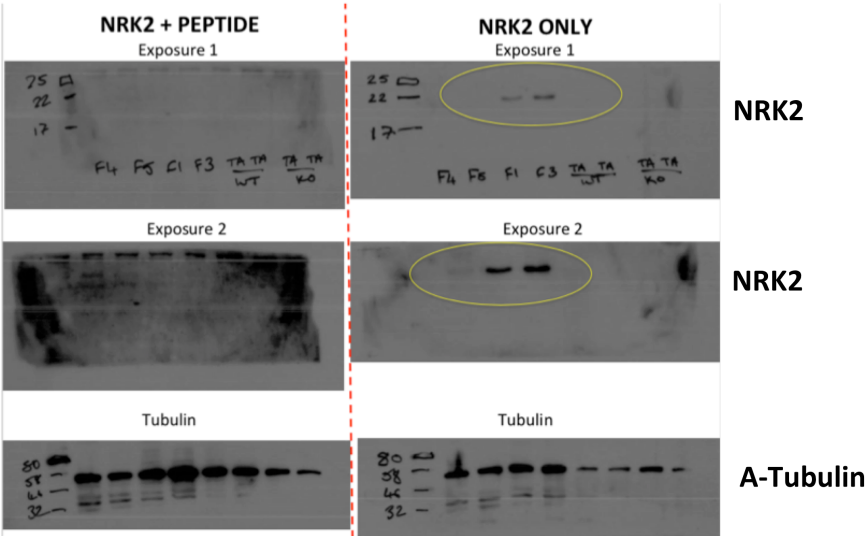

Supplement: Supplementary file 2 — Additional file 2: Figure S2. NRK2 detection with Western blot. (a) Western Blot of skeletal muscle lysates taken from TA muscle of Cre-negative NRK2 (equivalent to WT), skeletal muscle specific NRK2 overexpressing mice (NRK2ActaCre+/+), Wild type (WT) and NRK2 knockout (KO). NRK2 protein is evident at 22 kDa in all WT and transgenic lysates. It is absent in NRK2 KO tissue. (b) Western Blot of skeletal muscle lysates taken from WT(F4 & F5) and H6PD KO(F1 & F2) mice. Alongside are muscle lysates form WT and NRK2 knockout mice. The left panel shows blots incubated with the NRK2 antibody and the blocking peptide and results in no signal for NRK2 being obtained. Panel on the right shows blots incubated with NRK2 antibody, demonstrating bands at 22 kDa. [file 13395_2019_216_MOESM2_ESM.pdf]
